# Simulation of Human Movement in Zero Gravity

**DOI:** 10.3390/s24061770

**Published:** 2024-03-09

**Authors:** Adelina Bärligea, Kazunori Hase, Makoto Yoshida

**Affiliations:** 1 Department of Mechanical Systems Engineering, Tokyo Metropolitan University, 6-6 Asahigaoka, Hino 191-0065, Tokyo, Japan; kazunori.hase@tmu.ac.jp; 2 Department of Physics, Technical University of Munich, James-Franck-Straße 1, 85748 Garching, Germany

**Keywords:** body tracking, Azure Kinect, kinematic modeling, OpenSim, human spaceflight

## Abstract

In the era of expanding manned space missions, understanding the biomechanical impacts of zero gravity on human movement is pivotal. This study introduces a novel and cost-effective framework that demonstrates the application of Microsoft’s Azure Kinect body tracking technology as a motion input generator for subsequent OpenSim simulations in weightlessness. Testing rotations, locomotion, coordination, and martial arts movements, we validate the results’ realism under the constraints of angular and linear momentum conservation. While complex, full-body coordination tasks face limitations in a zero gravity environment, our findings suggest possible approaches to device-free exercise routines for astronauts and reveal insights into the feasibility of hand-to-hand combat in space. However, some challenges remain in distinguishing zero gravity effects in the simulations from discrepancies in the captured motion input or forward dynamics calculations, making a comprehensive validation difficult. The paper concludes by highlighting the framework’s practical potential for the future of space mission planning and related research endeavors, while also providing recommendations for further refinement.

## 1. Introduction

In the current era of surging manned spaceflight missions, particularly with the rise of commercial ventures, understanding the biomedical and physical impacts of a gravity-free environment on humans is increasingly crucial [1,2,3]. An essential aspect of this exploration lies in comprehending how weightlessness influences human movement and coordination. Previous research has, for instance, highlighted the dramatic consequences of weightlessness on orientation and posture, impacting the health and well-being of astronauts [4]. Given the prohibitive costs of conducting experiments in space, advancements in biomechanical simulations allow for more and more accurate replications of the physical behavior of the human body in various environments, making simulation tools a convenient alternative to real experiments [5].

This paper presents an innovative and straightforward framework for simulating motion in zero gravity by seamlessly integrating Microsoft’s Azure Kinect body tracking technology [6] into the biomechanical simulation tool OpenSim [7]. OpenSim, a freely available software package, is renowned for its ability to conduct efficient and accurate simulations of musculoskeletal dynamics and neuromuscular control [8,9]. Our approach introduces the novel concept of a human controller—utilizing a multi-camera setup to capture authentic motions from an actual person to generate realistic motion input. In contrast to traditional modeling approaches like those proposed by Kailai et al. [10] and Badler et al. [11,12], our framework aims to more closely replicate the intricacies and variations of human movement in zero gravity simulations. While traditional models often face computational and modeling limitations, our approach leverages the capabilities of a motion capture system, focusing on the potential rather than the constraints. By employing simple forward dynamic calculations to accurately translate the observed kinematics captured from recorded movements, we propose a cost-effective method that could be advantageous for experimental exploration.

To test and validate our framework, we investigated the consequences of linear and angular momentum conservation, evident in the absence of external forces or torques. In this context, we explored the possibility of realizing rotations, locomotion, and full-body coordination tasks in a weightless, ground-free environment. Moreover, we pose the intriguing question: How would hand-to-hand combat manifest in space? This query will likely gain relevancy in the context of burgeoning space tourism or even population prospects. Drawing on the martial arts expertise of one of the authors, specifically in Karate, we conduct simulations on various combat-related movements to provide insights into this question. Serving as a proof of concept, our results and their possible implications aim to foster future research in the domains of human space flight and space medicine, such as finding novel exercise routines for astronauts.

## 2. Methods

In the quest to accurately reproduce human motion in the unique setting of zero gravity, our methodology relies on a human-controlled input derived from a recorded video of human movement. Utilizing Microsoft Azure Kinect DK’s advanced depth sensing technology, we made a recording of the specific target movement. This data was then converted into a digital motion format using the body tracking capabilities of Azure Kinect’s software development kit [6]. Subsequently, the motion input could be transformed to its virtual representation within OpenSim [7] (API version 4.4). In a ground-free environment, zero gravity conditions were applied using OpenSim’s inherent functions and implementing proportional-derivative (PD) control for precise forward dynamics calculations. The subsequent sections will explain each step of this progressive methodology. The complete C++ code for implementing our framework will be made available as Supplementary Materials.

### 2.1. Azure Kinect Technology

The video recordings used for the motion input employed Microsoft’s Azure Kinect DK devices (from Microsoft Corporation, Redmond, Washington, US) utilizing their 1 MP depth camera. Emitting modulated light in the near-infrared spectrum onto the scene, it records the time taken for the light to travel from the camera to the scene and back. The recorded data is then processed to generate a depth map, providing z values for each pixel. Details on the operation mode of the cameras employed for this research can be found in Table 1.

**Figure 1:**
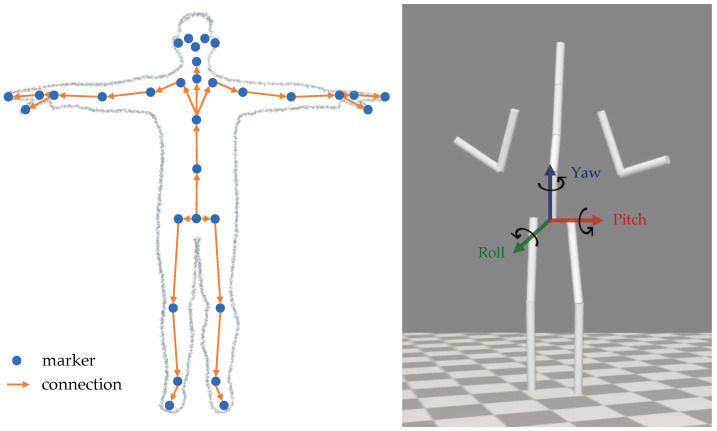
**Left**: Joint markers (points) from the Azure Kinect body tracking kit [16]. The connections (arrows) each link one parent joint with a child joint. **Right**: Our virtual multi-body model, with a segmentation (indicated by white cylinders) aligning the marker positions on the left. To facilitate orientation in later motion analysis, the figure displays the yaw, roll, and pitch rotational axes originating from the pelvis segment.

Azure Kinect complements these sensor capabilities with a 3D body tracking kit [14], allowing for precise motion capture. Therein, estimates of the position and orientation of 32 joint markers (as displayed on the left of Figure 1) are provided relative to the global depth sensor frame of reference. Each of them defines their unique right-handed coordinate system, all of which are absolute coordinate systems within the depth camera’s 3D depth map. The resulting skeletal structure forms a comprehensive representation of the human body’s articulation [15].

### 2.2. Multi-Camera Motion Capturing

To enable the capture of more intricate and spatially demanding motions, a setup with multiple synchronized Azure Kinect DK devices was employed. These cameras were strategically positioned to record different angles of the performed movements (see Section 2.6). This approach aimed to increase the effective camera coverage within the designated space, as a second camera can be used to make up for potential occlusions and errors in the tracked joint positions of the first. In the end, this should lead to a more accurate and faithful reproduction of the recorded motion [17,18].

However, the official software development kit [6] provided by Microsoft does not offer a specific routine for body tracking using multiple synchronized cameras. Therefore, we took the initiative to create a customized setup and calibration routine, which will be presented in the following. Note, that the experiments detailed in this paper relied on the use of two Azure Kinect DK devices, but the established routine can be seamlessly extended to more sensors if needed (see Section 2.2.1 for more detail).

#### 2.2.1. Synchronize Recordings

To connect both devices, we employed a “daisy-chain” configuration [19], in which the master device provides a triggering signal for the subordinate devices. When multiple depth cameras capture overlapping fields of view, each camera must image its respective laser independently. In order to mitigate interference between the lasers, the camera captures were temporally offset by 160 μs. Although the laser’s actual pulse width is 125 μs, the handbook [19] recommends a larger offset to provide additional margin. By employing a delay of 160 μs, synchronization of up to nine additional depth cameras (making ten in total) is achievable by interleaving their exposure periods (c.f. Table 1). This arrangement ensures that each camera’s laser is activated only during the idle periods of the others, effectively minimizing cross-interference.

For the synchronization of the captures itself, the *MultiDeviceCapturer* class specified in the *green_screen* example of the Azure Kinect software kit [6] was deployed and embedded into the recording setup. In the following, the master device is denoted by 1, while the subordinate device is denoted as camera 2.

#### 2.2.2. Transform Coordinate Systems

**Figure 2:**
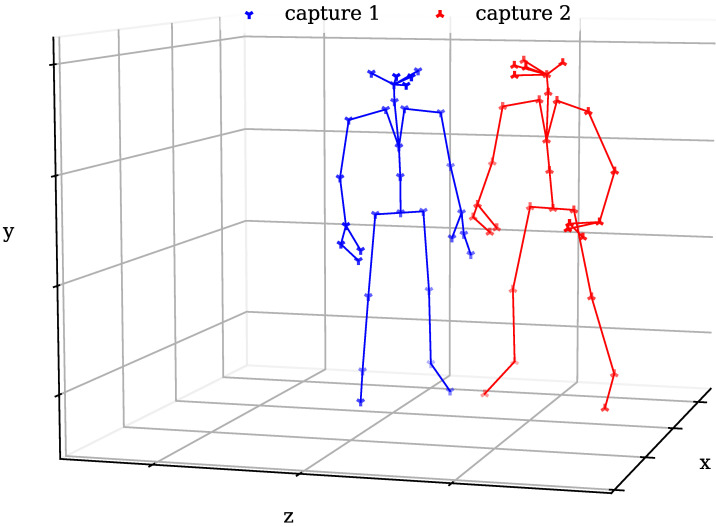
3D marker skeletons as captured from two synchronized cameras. Before coordinate transformation, the assigned positions for each marker of the two captures do not overlap.

After having synchronized recordings of a motion, the Azure Kinect body tracking developer kit [14] can be used to capture the location and orientation of joint markers (as displayed on the left of Figure 1) for each frame. However, this capturing is only done with respect to the coordinate system used by each recording camera. Figure 2 shows two captured body skeletons based on the marker positions in their respective coordinate systems. It is clear from this picture that these locations do not match. Therefore, it is necessary to transform the captured coordinates of one camera to match the coordinate system of the other one.

Based on code by Jeon et al. [20], the authors utilized Arun’s method for 3D registration [21] to achieve this transformation. It essentially describes a non-iterative least-squares approach that matches two sets of 3D points via linear transformations (rotation and translation) for a given correspondence between the points.

#### 2.2.3. Merge Motions

Once both motions are provided in a common coordinate system, they can contribute to creating one final, more confident capture. The Azure Kinect body tracking kit [14] supplies confidence level indicators in ordinal scale for each tracked marker location. These alphabetical indicators were arbitrarily assigned to numerical values using the mapping:
confidence levelNONELOWMEDIUMassigned value012

This established them as weights $w_{i}$ in the merging procedure, as follows: If exactly one of the capture’s markers had a confidence level of 0, the location of the non-zero confidence capture was used. Otherwise, the following formula for a weighted mean was applied:

(1) $$r_{i}=\frac{w_{1,i}\;r_{1,i}+w_{2,i}\;r_{2,i}}{w_{1,i}+w_{2,i}}$$

where $r_{i}$ is the *i*-th linear coordinate of the merged marker position r, while $r_{1}$ and $r_{2}$ stand for the captured position of the recording of camera 1, and the transformed positions of camera 2 (fitted to the first camera’s global coordinate system), respectively. Note, that in the rare cases in which Arun’s method failed to correctly transform the coordinates $r_{2}$ of the second camera, all confidence indicators $w_{2}$ of this capture were automatically set to 0, such that it was ignored altogether, erasing possible jumps in the merged motion capture.

**Figure 3:**
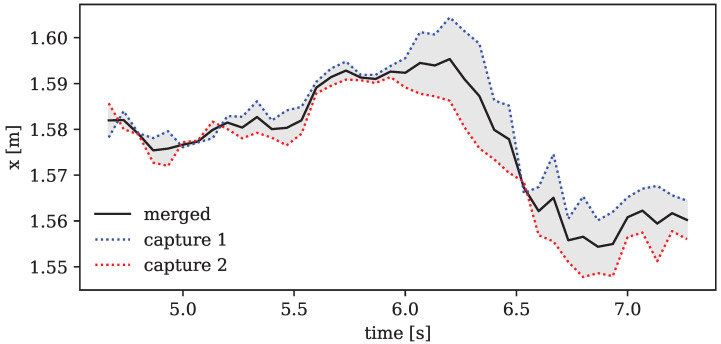
Motion excerpt of one exemplary marker in x-direction. The dotted curves show the transformed linear coordinates of the two captures. The solid line is the merged solution after applying Equation (1).

The result of this merging routine can be seen in Figure 3, which shows an exemplary excerpt of one marker’s coordinate track over time. The combined motion seems to successfully correct some incongruities and outliers of each of the captures by interpolating between them.

### 2.3. Noise Filtering

Even when employing multiple cameras, the accurate recognition of joint markers may remain challenging, resulting in possible noise and outliers in the tracked motion curves. Such discrepancies can lead to abrupt transitions in the simulated motion, potentially undermining or disabling the precision of zero gravity calculations. Consequently, a smoothing routine, grounded in generalized cross-validation splines and integrated into the OpenSim API [7] following Woltring [22], was applied to the temporal evolution of captured marker positions.

**Figure 4:**
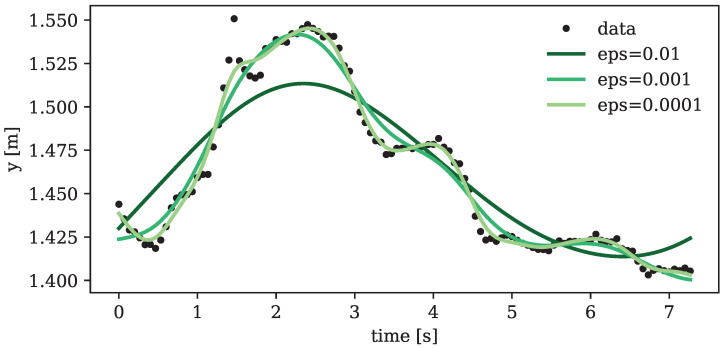
Generalized cross-validation splines (cubic) applied to the motion of an exemplary marker in y-direction. Black dots represent the actual recorded motion, while the three curves depict splines with varying error variance (denoted by “eps”), each demonstrating a different level of smoothing.

To avoid the risk of overfitting, cubic splines were chosen and systematically tested across various error variances inherent in the data. Figure 4 illustrates the fitted splines for different error variances. Striking a balance between degrees of smoothing and avoiding excessive underfitting, a default error variance on the order of $\mathrm{eps}=10^{-4}$ emerged as the optimal choice. This parameter, however, underwent subsequent adjustments for each specific target motion based on the observed erratic behavior of the final capture.

### 2.4. Human Model Configuration

Concluding the preprocessing pipeline, the construction of a human model is necessary to align the captured marker locations with the natural joints of the human body. To achieve this, we used the biomechanical simulation framework offered by OpenSim [7]. Our model is based on the comprehensive 3D multi-body model developed by Hase and Yamazaki [23], which was optimized for human gait simulation [24]. To enhance its suitability to the specific movements and capturing procedure undertaken in this study, several modifications were implemented:

The segmentation of the spine was changed to correctly match the location of the joint markers offered in the Azure Kinect body tracking kit [14] (see Figure 1). To simplify calculations further, the feet were removed from the model due to their irrelevancy for the target input motions, however, adding their body weight to the lower legs. The lengths and weights of all individual segments were adjusted to exactly fit those of the recorded person. To determine the mass of each body segment, established segment ratios were applied to the total body weight, thereby distributing the overall mass in accordance with the specific proportion each segment contributes to the full body mass. These ratios and the respective location of the center of masses for all segments were taken from Table 4.1 by Winter [25]. A missing rotational degree of freedom was added to the shoulder joints, a feature omitted in the original model due to its negligible role in human gait simulation. Furthermore, the passive moments of each joint degree of freedom (“coordinate limit force” in OpenSim) were changed from Davy and Audu’s model [26] as used by Hase and Yamazaki [23] to more generally valid empirical values so that the model would be suitable for a larger variety of possible human movements.

The right side of Figure 1 shows the final 12-link-segment model (weight: 63 kg, height: 173 cm), where cylinders visualize each body segment for simplicity. For applying the captured motion of marker coordinates to this model, we used a simple inverse kinematics calculation, as is part of the OpenSim framework. This results in a generalized representation of the motion, manifested in the angles of each joint degree of freedom of the model at each time step.

### 2.5. Zero Gravity Simulation

With the model and motion capturing routine now established and fine-tuned to generate a reliable digital representation of the motion kinematics based on the recordings, we can delve into the intricacies of the zero gravity simulation.

The simulation of human movement in a new gravitational environment essentially involves calculating a kinematic forward solution with a modified gravitational force vector acting downward onto the center of mass of each body segment. In the case of zero gravity, this force is omitted, a parameter conveniently adjustable within OpenSim. To simplify calculations and align with scientific interests, we placed the body in a ground- (and wall-) less environment by imposing a constant vertical offset onto every captured marker location, allowing us to bypass the modeling of ground reaction forces.

In our study, we utilized a proportional-derivative (PD) controller to model the forward dynamic model solution. At every time step of the motion, the PD control aims to correct the error between the measured process variable (input motion coordinates $\theta_{\mathrm{input}}$ and $\omega_{\mathrm{input}}$) and the desired set point (output motion coordinates θ and ω). While the proportional control (P) minimizes fluctuations in the process variable (angular position), the derivative control (D) minimizes changes in the error variable (angular velocity), maintaining system stability.

To these ends, the PD controller applies a constant torque (the control value)

(2) $$\tau =-\;k_{\theta }\;\cdot \;\underset{\mathrm{position}\;\mathrm{error}}{\underset{︸}{\left(\theta -\theta_{\mathrm{input}}\right)}}-k_{\omega }\;\cdot \;\underset{\mathrm{velocity}\;\mathrm{error}}{\underset{︸}{\left(\omega -\omega_{\mathrm{input}}\right)}}$$

to each joint of the model, adjusted per time step $\Delta t=\frac{1}{3000}$s (corresponding to $\frac{1}{100}$ of the frame rate) to minimize the errors. For the positional and velocity error, the coefficients $k_{\theta }$ and $k_{\omega }$ are gain factors, effectively translating their physical dimensions into torques for joint actuation. As the input motion data, formatted for OpenSim, originally contained only positional information (joint angles at each time step) with no direct velocity data, we applied quintic spline interpolation to the angle data to generate the positions $\theta_{\mathrm{input}}$ and velocities $\omega_{\mathrm{input}}$, the latter derived from the spline’s first-order derivative. This procedure provides smooth, continuous estimates of the joint angles and velocities at a resolution finer than the original data, making it suitable as input for the PD control calculations. Recognizing the limitations of our input data, which did not contain velocity information, the output variable ω was set to zero in our simulation, thus aligning OpenSim’s model state for the output motion with the available data. Note that while this decision simplifies the computational model, it changes the interpretation of $k_{\omega }$ and therefore the role of velocity feedback in our control mechanism, shifting the focus towards its adaptability to positional data.

**Figure 5:**
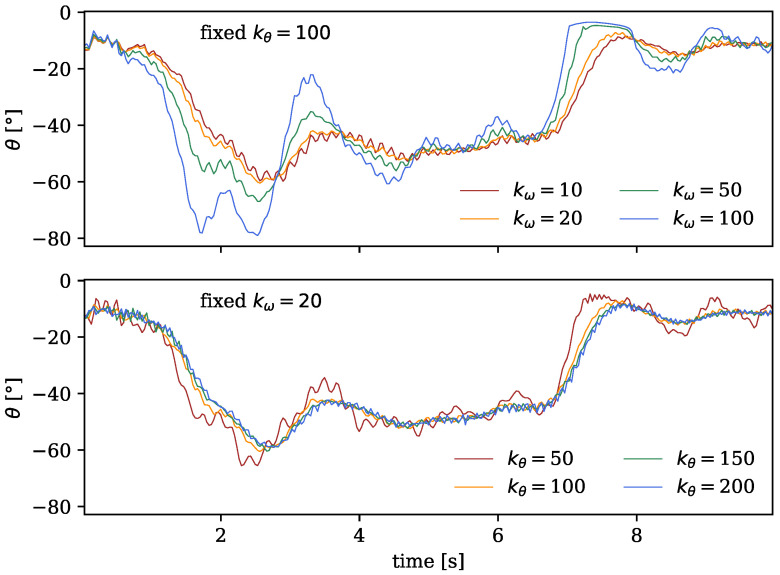
Right knee joint angles over time, with the negative y-direction corresponding to an increase in flexion. Impact of velocity error gain coefficients $k_{\omega }$ (**top**, for $k_{\theta }=100$ fixed) and of position error gain coefficients $k_{\theta }$ (**bottom**, for $k_{\omega }=20$ fixed) on stability of output motion curve.

As evident in Equation (2), the extent of both P and D control is governed by the error gain coefficients $k_{\theta }$ and $k_{\omega }$. To determine the optimal parameters for the forward dynamics calculation, we conducted a trial-and-error analysis by systematically exploring different coefficients and visually investigating their impact on the stability of the generated output motion. Figure 5 shows the explored motion curves of the model’s right knee joint, which when focussing on stability are exemplary for the motion of the rest of the body, as the outer extremities (such as the lower legs) are expected to be most affected by instabilities in the forward simulations. In the upper plot, $k_{\theta }$ was set to the arbitral value of 100, which is the default used in a similar routine of the OpenSim API [7], while the velocity gain $k_{\omega }$ was varied. The corresponding curves in the top of Figure 5 visually reveal that the most confined and least shaky motion occurred with a gain of $k_{\omega }=20$. The same procedure was employed in the lower plot, maintaining $k_{\omega }=20$ constant and varying the positional error gain $k_{\theta }$, which resulted in $k_{\theta }=100$ or $k_{\theta }=150$ as the visually most stable motion curves in Figure 5. Consequently, in the final simulation routine, we adopted $k_{\omega }=20$ and $k_{\theta }=100$ as the default parameters.

### 2.6. Experimental Setup

**Figure 6:**
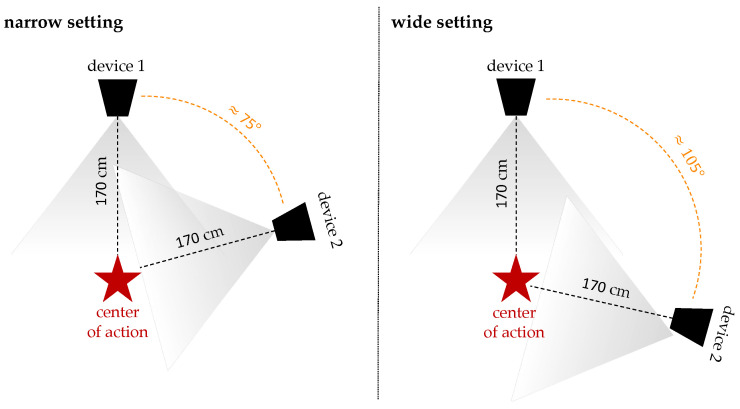
Schematic illustration of the two measurement setups used for recording the input motions. **Left**: narrow setting with the cameras positioned at an angle of approx. 75°. **Right**: wide setting with a separation angle of approx. 105°.

For the experiment, we recorded a set of 18 movements, each for 15 s, using two Azure Kinect DK devices in the distinct setups illustrated in Figure 6. The devices were interlinked and synchronized for both setups following the procedure outlined in Section 2.2. The reason behind employing these two settings was to capture multiple different angles of the same motion, aiming to enhance the likelihood of accurate marker tracking, which should, in turn, increase the probability of a faithful motion output. Depending on the executed motion, one of these camera settings usually demonstrated better performance in capturing, which is why for the final evaluation only the more accurate dataset was selected for each corresponding motion. All movements were carefully selected to address one or more of the primary research questions defined in Section 1. A summary of the recorded movements and their respective contributions to the prevailing research questions is presented in Table 2 for reference in subsequent evaluations.

**Figure 7:**
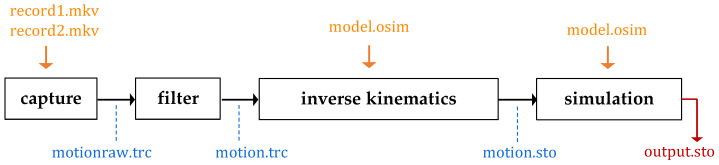
Visualization of the workflow for processing the recordings, to the generation of a simulated output motion. Each black box represents an independent routine in the progress, taking input data (upper and lower band) and producing a processed file (lower band).

A total of 18 (motions) ·2 (settings) =36 pairs of recordings were taken as input for the experiments. All of these recordings went through the preprocessing procedure outlined above and summarized in Figure 7:

First, the **capture** routine utilizes the Azure Kinect body tracking tool kit [14] for the two video files, *record1.mkv* and *record2.mkv*, and consolidates the two tracked motions into a unified capture file, *motionraw.trc*. Subsequently, the **filter** routine applies a spline smoothing algorithm to the coordinate tracks, producing a noise-reduced motion capture, *motion.trc*. Following this, the **inverse kinematics** routine translates the coordinates of the tracked markers into joint angles of the model (derived from *model.osim*). This process results in a motion file, *motion.sto*, tailored for display in OpenSim. Finally, the sequence finishes with the **simulation** of the motion in zero gravity, performing a forward dynamics calculation of the input motion without external forces, resulting in the final *output.sto* file.

## 3. Results

In this section, we aim to present and analyze the results of the 18 simulated motions outlined in Table 2. Each motion will be referred to by its assigned index throughout the subsequent analysis.

As introduced earlier, we assess the validity of our simulations by examining two fundamental conservation laws applicable in a free (wall- and ground-less), zero gravity setting: the conservation of linear momentum when no external forces act on the body and the conservation of angular momentum in the absence of external torques. Both principles restrict the potential movement of a human body’s center of mass and its ability to rotate. To scrutinize these conditions, we conducted recordings of various motions involving rotational elements (3, 4, 5, 6, 13, 14) and locomotion (6, 16, 17, 18), subjecting them to our simulation framework for analysis. All these motions were started in a stationary standing position to ensure that the simulated body’s initial total linear and angular momentum would be zero.

### 3.1. Rotation

Initially, we assessed rotations of the arms around the lateral axis while maintaining a fixed stance: once with the arms in a more vertical position (motion 3) and once in a more horizontal position (motion 4). Both instances exhibited observable changes of the body’s inclination in the zero gravity simulation.

**Figure 8:**
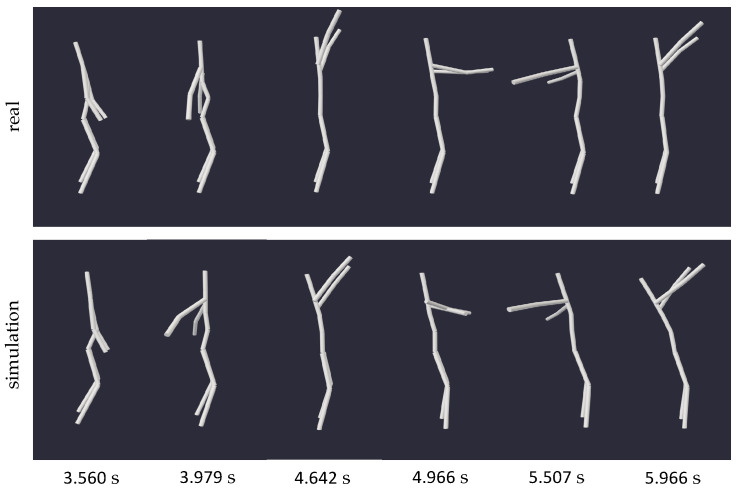
Series of snapshots capturing motion 3 (rotating arms in fixed stance) and its zero gravity simulation at specific times. The alignment of the two moving models from equal viewing angles facilitates a comparison of the bodies’ inclination throughout the motion.

**Figure 9:**
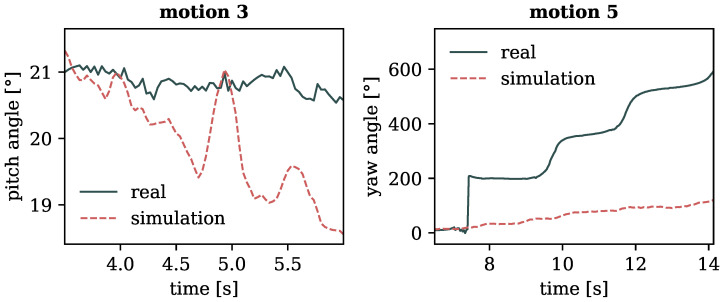
**Left**: Angle of pitch rotation of the pelvis over time for motion 3 (rotating arms in fixed stance). **Right**: Angle of yaw rotation of the pelvis over time for motion 5 (stepwise full-body rotations). Generally, rotation angles are in counter-clockwise direction around their respective base axis. Angles of 0° represent a wholly upright standing position.

In Figure 8, a series of images depicts an excerpt of these arm rotations from motion 3, facilitating a comparison between the inclination of the body in a gravity-exposed state and a weightless environment. While in the former, the body remained upright, in the zero gravity scenario, it seems to lean increasingly backward, suggesting the possibility of starting a rotation around the lateral axis. The same observation can be made when examining the angle of the pitch rotation of the pelvis (approximately representing the body’s center), depicted on the left side of Figure 9. While its value in the gravity-dependent motion remains relatively constant (upright position), the weightless scenario exhibits a notable change in the body’s orientation around the lateral axis, expressed by a decreasing pitch angle of the pelvis.

Note that the rotational angles of the pelvis—yaw and pitch as outlined in Figure 1 and depicted in Figure 9—are not governed by PD control, as defined in Equation (2). This exception arises because the lower end of the pelvis does not simulate an actual bodily joint, but rather functions as a link between the body and the surrounding space.

The observable change between actual and weightless movement in Figure 9 therefore arises primarily from the differences in how angular velocity, initiated by the arm movement, is managed. In the real, gravity-affected setting, this change of momentum is counterbalanced by ground reaction forces. However, in the simulation, which omits any external forces, the same angular velocity can impart a rotational motion to the weightless body model.

Furthermore, we explored full-body rotations around the vertical axis by employing broad sweeping movements of the arms in motion 5. In the typical gravitational setting, these movements manifested in stepwise rotations of 180° each. However, in the zero gravity scenario, the expansive arm movements induced a noticeable but more subtle rotation of about 100° in total. This observation is illustrated on the right side of Figure 9, displaying the corresponding yaw angles for both motions. The constrained rotation in the weightless movement, as compared to its motion input, can be attributed to the fact that the latter utilized not only the arms to achieve the twisting, but also partly relied on ground reaction forces, which were absent in the weightless setting of the former.

The consequent ability to induce rotation might appear to contradict our initial requirement for a constant angular momentum of the body, defined as the product of angular velocity and moment of inertia. However, Frohlich [27] clarifies that the law of angular momentum conservation is deceptive: it does not actually necessitate a constant angular velocity. Both the angular velocity and moment of inertia can vary inversely as long as they maintain a constant product. Frohlich’s study on the physics of diving [27], closely related to that of astronaut motion due to the absence of torques, even suggests the feasibility of zero-angular momentum twists. The resulting capability to reorient the body in a weightless environment, even from a motionless starting position, also aligns with observations mentioned by Badler et al. [11].

### 3.2. Locomotion

The law of linear momentum conservation, which is the product of a body’s mass and its linear velocity, has a different implication for the motions’ realizability in zero gravity. As the mass of a body always remains constant, the conservation of linear momentum postulates a constant linear velocity, with both the velocity and momentum vectors consistently parallel. In the absence of external forces, as simulated in our ground-less zero gravity environment, this implies that the net movement of the body’s center of mass should be zero, essentially forbidding any form of locomotion or gait.

**Figure 10:**
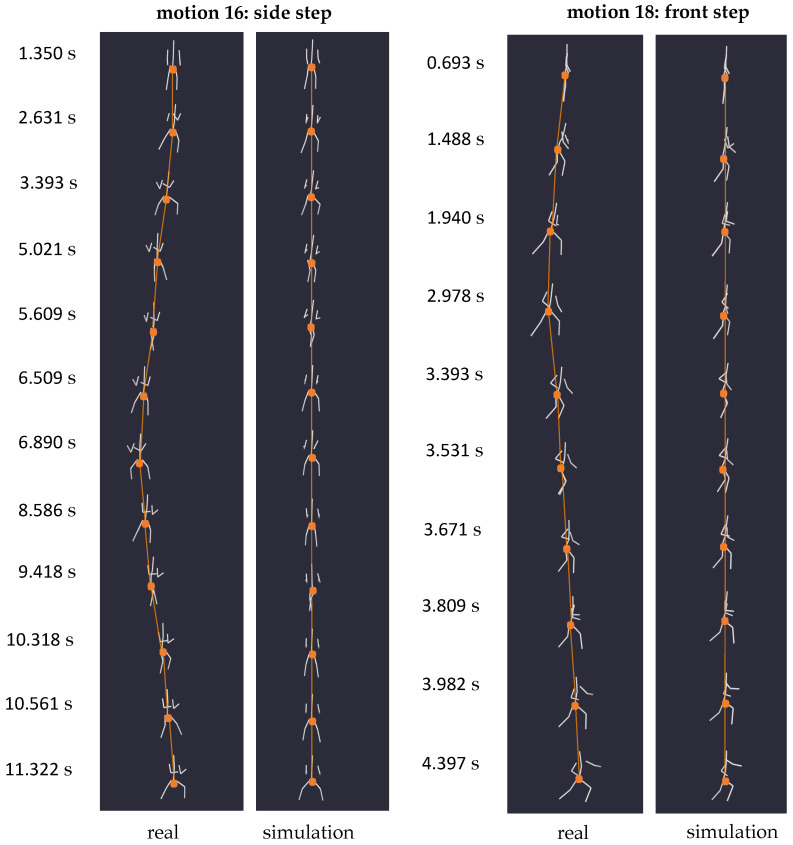
Series of snapshots capturing motion 16 (side step) on the (**left**) and motion 18 (front step) on the (**right**) at specific times, each with their respective zero gravity simulation. The alignment of the two moving models from equal viewing angles facilitates a comparison of the location of the bodies’ centroids throughout the motion, indicated by dots and connections.

To substantiate this phenomenon, we recorded three different step types (16, 17, 18) and one jump with positional change (6). In each instance, the simulation yielded a scenario where the centroid of the human body remained unchanged, with only partial movements of the body’s extremities reproduced. Figure 10 illustrates the simulation results of two exemplary step motions, one to the side (16) and one to the front (18), with the location of the pelvis marked by dots. While in reality, these steps induced a clear change in position within the specified space, the weightless environment caused the body’s centroid to remain stationary, as evidenced by the immobile pelvis of the simulated body. This outcome aligns with similar experiments conducted by Kailai et al. [10], further validating our simulation results.

### 3.3. Coordination

Now that we have confirmed the physical validity of our simulation framework in terms of its conformity with essential conservation laws, we leverage our tool to investigate the feasibility of executing full-body coordination tasks in a weightless environment. Our experimental set comprised seven motions (5, 6, 11, 12, 13, 14) designed to pose challenges regarding full-body coordination.

**Figure 11:**
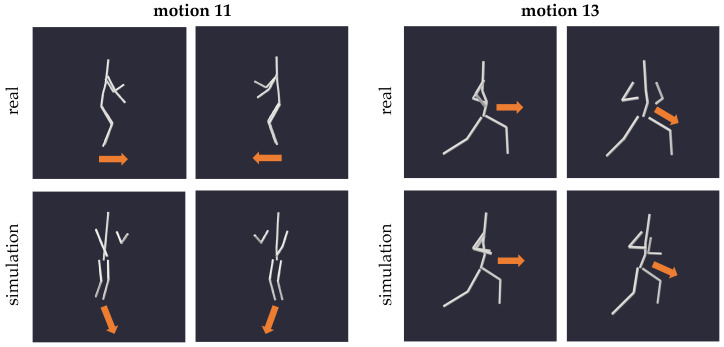
Exemplary snapshots of specific times of motion 11 (full-body turns) and 13 (hip rotation) and their respective zero gravity simulations, viewed from the same angle. For a better comparison of the bodies’ postures, the orientation of the pelvis segment is indicated by arrows.

Adhering to the conservation laws clarified earlier, our simulations unveiled constraints in the mobility of the body centroid and its rotational capabilities when exposed to zero gravity. Figure 11 depicts excerpts of motion 11 featuring full-body rotations, and motion 13 featuring hip rotations, both exhibiting limited reproducibility in zero gravity due to the absence of external forces and torques. As indicated by the arrows in Figure 11, the changes in the orientation of the body and hip were not exactly reproducible in the weightless setting; instead, only subtle movements are discernible.

**Figure 12:**
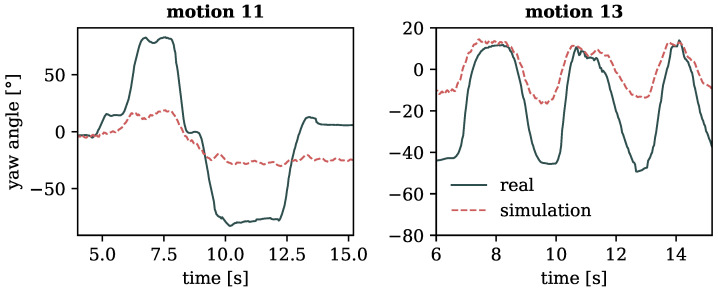
Angle of yaw rotation of the pelvis over time for motions 11 (full-body turns) on the (**left**) and 13 (hip rotation) on the (**right**).

These constraints are also evident when examining the angle of yaw rotation of the model, as depicted in Figure 12. In both motions 11 and 13, the rotation angle around the vertical axis appears as a damped version of the input motion, indicating the limited possibility to turn the body in a weighless setting with no external forces. Note that the slight offset in trajectories on the right plot of Figure 12 likely arises from variations in stance between the actual motion and its zero gravity counterpart (cf. Figure 11), which is due to the lack of ground reaction in the simulated environment.

**Figure 13:**
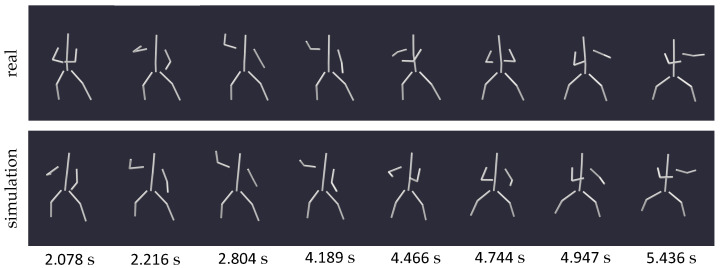
Series of snapshots capturing motion 12 (full-body coordination task) and its zero gravity simulation at specific times. The alignment of the two moving models from equal viewing angles facilitates a comparison of the bodies’ postures throughout the motion.

Despite the body centroid’s immobility, the body’s extremities adeptly replicated the target motions, successfully executing the required coordination tasks. This means that coordination movements with changes in orientation (5, 6, 11, 13, 14) induced by ground reaction forces were unattainable in our simulated zero gravity environment, except for the isolated movement of extremities. Conversely, motions executed in a fixed position and orientation demonstrated remarkable reproducibility in the weightless setting, yielding results comparable to those observed in gravity. Motion 12, for instance, as depicted in Figure 13, was nearly perfectly reproducible.

**Figure 14:**
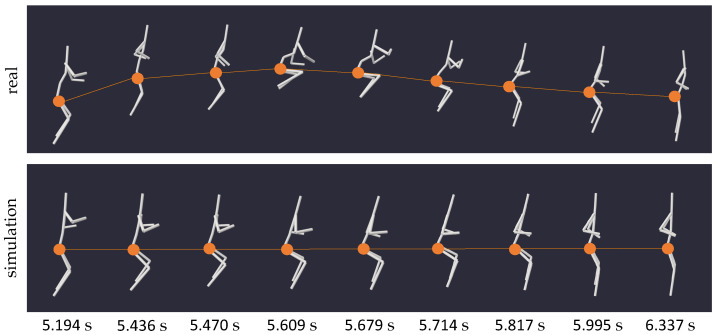
Series of snapshots capturing motion 15 (two-legged jumps on the spot) and its zero gravity simulation at specific times. The alignment of the two moving models from equal viewing angles facilitates a comparison of the location of the bodies’ centroids throughout the motion, indicated by dots and connections.

A coordination task that proved particularly intriguing in our simulation tool was a two-legged jump on the spot (motion 15), a maneuver inherently reliant on ground reaction forces. Conceptually, jumping resembles a form of vertical locomotion, and thus, we expect comparable results to those discussed in the previous paragraph. Figure 14 visualizes the actual motion and its zero gravity simulation, with the bodies’ hip joints marked by dots in both instances. While on Earth, the centroid of the human body would ascend and descend during a jump, it remains fixed at one height in a weightless environment due to momentum conservation. The resulting motion appears more akin to a floating squat.

**Figure 15:**
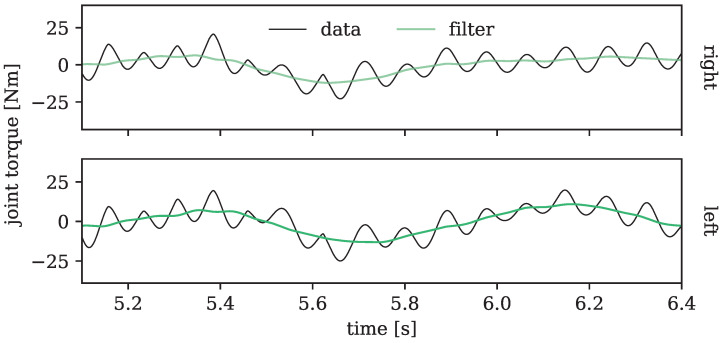
Joint torques of the left (**top** plot) and right (**bottom** plot) hip joint of the zero gravity simulation of motion 15 (two-legged jump on the spot) over time, with the positive y-direction corresponding to an increase in flexion. The dark, solid line shows the actual torque values as outputted by the simulation, while the smooth line represents a filtered version of the curve, better presenting the overall trend of the shaky output.

In Figure 15, we examined the hip torques for extension and flexion during the motion excerpt presented in Figure 14. In addition, a filtered version of the curve was plotted to show its overall trend more clearly. Despite the noticeable shakiness, the curve unmistakably displays a raising and lowering in joint torque, suggesting a clear pattern of muscle activity during the weightless jump, reminiscent of a floating squat. These findings suggest that our framework, coupled with the analysis of joint torques, could hold significance in formulating device-free, unassisted exercise routines for astronauts in space.

### 3.4. Martial Arts

As mentioned in the introduction, our investigation extends to the weightless adaptations of martial art movements, seeking insights into the potential dimensions of hand-to-hand combat in a microgravity environment. Essentially, martial art movements just represent a special subset of full-body coordination tasks; thus, the outcomes of this section are anticipated to align with the findings discussed in the previous paragraph. However, this exploration emphasizes the precision of executed motions, recognizing the critical relevance of the exact placement of body parts in combat scenarios. To address this, we examined both attacks (7, 8, 9, 10) and defensive maneuvers (1, 2).

**Figure 16:**
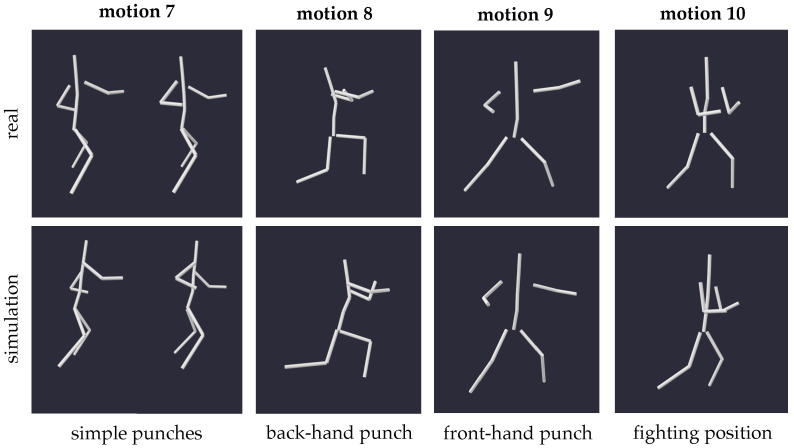
Exemplary snapshots of specific times of four typical fighting motions with their respective zero gravity simulations, viewed from the same angle to better compare the bodies’ exact postures.

Figure 16 provides an overview of the simulation outputs for various movements, such as punches, which are pertinent to unarmed combat. It is evident from these depictions that while the simulated motion closely resembles the input, it is not very exact. This observed discrepancy is consistent across the experiment’s defensive and offensive motions.

**Figure 17:**
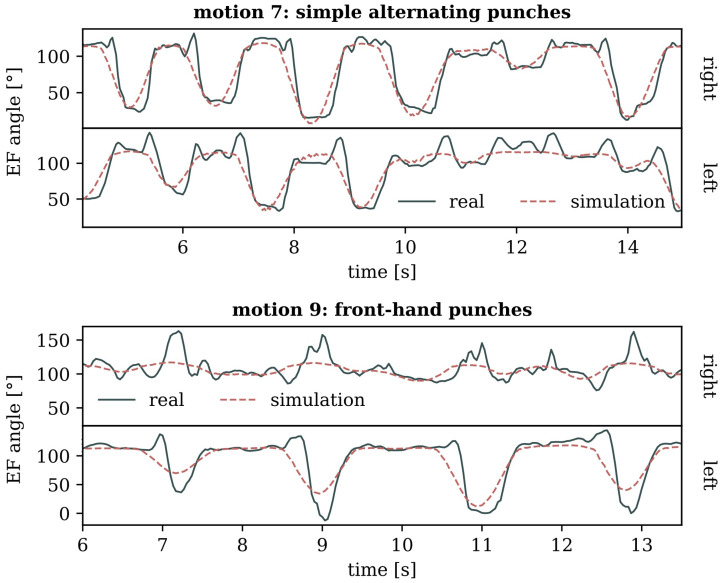
Joint angles of left and right elbow extension and flexion (EF) during two punching motions (top: simple alternating punches, bottom: front-hand punches), with a positive slope corresponding to flexion. The extremes of the curves mark the placement of individual punches.

This circumstance was further analyzed by comparing the exact flexion and extension angles of the elbows during punching motions, both for the tracked input and the simulation. In Figure 17, the trajectories of the model’s elbow joint angle values display the punch placements in the executed motion by the extremes of the curves. Across all plots in Figure 17, the simulated motion consistently falls short of reaching the same extreme values as the target, indicating a limitation in the weightless body’s ability to extend and flex the elbow while punching. Upon close examination of Figure 17, however, it becomes evident that all simulated motion curves resemble a smoothed version of the input. This resemblance suggests that the lack of precision might also be attributed to the controller algorithm employed in the forward dynamics calculations of the zero gravity simulations, potentially limiting our ability to draw definitive physical conclusions from the observed discrepancies. For a more thorough discussion on this issue, refer to Section 4.1.

## 4. Discussion

Having presented the simulation results, we now turn to focus on the methodology itself, highlighting notable limitations and challenges encountered during the evaluation process. Our simulation framework relies on three key components: the preprocessing procedure for generating the motion input out of Azure Kinect DK recordings, the PD control algorithm regulating the forward dynamics calculations, and the OpenSim model representing the moving body. Each of these components, detailed in Section 2, introduces potential sources of error in the simulation output.

### 4.1. PD Control

As detailed in Section 2 and illustrated in Figure 5, the control algorithm, responsible for regulating the forward kinematics calculation of the simulation, could sometimes introduce significant shivering in the output motion. This not only holds the potential to compromise the precision of motion data, as evidenced in Figure 17, but can also filter out potentially crucial aspects of motions, especially parts that are too subtle or fast, rendering them unaccounted for in the simulation.

**Figure 18:**
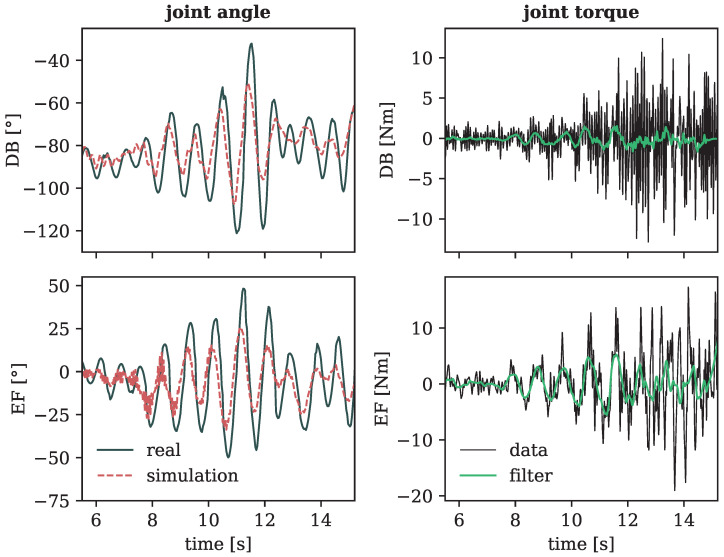
Joint angles (**left** side) and torques (**right** side) of the left shoulder during motion 4 (rotating arms) in the two degrees of freedom (DB: adduction-abduction and EF: extension-flexion), with a positive slope corresponding to abduction (**top**) and flexion (**bottom**). The right plots show the simulated torques of the forward dynamics calculations, with a smoother filtered line to better indicate the overall trend of the shaky output.

Figure 18 displays the joint angles and torques of the left shoulder during multiple pitch rotations of the arms (motion 4), with each rotary movement in a different inclination of the arms that altered the total diameter of rotation. This example shows a notable difficulty in accurately capturing subtle aspects of the motion, such as small circles made by the arms extended almost completely to the side, as depicted at the beginning of the curves in Figure 18. Moreover, on the right side of the plot, depicting the joint torques of the forward simulated motion (see Equation (2)), it is evident that these were nearly zero at the initiation of the motion despite a small-scale movement occurring in reality.

The authors acknowledge that the persistent issue of shivering, which is evident across all simulations (cf. Figure 5, Figure 15 and Figure 18), is likely due to our simplification in the implementation of the PD controller outlined in Section 2.5. The approach of setting the output velocity ω in Equation (2) to zero may inadvertently lead to positive feedback. While negative feedback typically fosters system stability by promoting convergence, positive feedback by its nature tends to amplify deviations, potentially increasing instability in the system output. Therefore, one possible improvement could be to approximate the angular velocities of the joints by finite differences. Implementing this correction may address accuracy issues in the simulated martial arts movements and likely mitigate the observed shakiness in all simulations. However, as these adjustments, while valuable, don’t detract from the core findings and conclusions of our study, refinement of this aspect is deferred to future research.

### 4.2. Human Model

The simulated motion output also depended on the employed OpenSim model utilized for the kinematics calculations (cf. Figure 7). As detailed in Section 2.4, the human model’s freedom of movement is implemented by applying passive moments to limit the joint degrees of freedom. However, these imposed restrictions of movement would cause challenges for the simulations. In cases where the input motion reached or exceeded the implemented coordinate limits of the joints, often due to a remaining imprecision in the capturing process, the simulation faced significant difficulties in interpreting and dealing with the given input, often resulting in failure. This emphasizes the crucial need for both an accurate model composition and a faithful capture to ensure the success of a simulation.

### 4.3. Preprocessing

Despite efforts to improve the stability and realism of the captured motion, instances of unstable data persisted, especially in motions involving changes in body orientation. Such input, often characterized by abrupt jumps, could lead to a time-out and failure of the subsequent simulation. Therefore, enhancements in the preprocessing phase could ensure a more reliable and stable presentation of the target motion to be simulated. We propose the following improvements:

- Use more than two cameras in the setup to achieve an even broader coverage of captured joint markers.
- Additionally incorporate the orientation measurements of joint markers obtained from the Azure Kinect body tracking software into the inverse kinematics calculation to elevate the accuracy of motion input.
- Investigate new algorithms, like those discussed by Eggert et al. [28], for transforming the coordinate systems of motion captures to address occasional failures in Arun’s method [21]. Alternatively, consider adopting one of the reliable calibration routines proposed by Romeo et al. [29].
- Improve the merging of captures by introducing a new measure for judging the consistency of tracked motion curves, beyond relying solely on confidence level indicators.
- Investigate alternative methods for addressing jumpiness of the motion input, such as categorizing and removing outliers before applying spline smoothing, as the current approach only mitigates their impact. Alternatively, consider utilizing a different filter function provided by OpenSim.

In addition to the above ideas, the low sampling rate of 30 FPS of our recording devices (see Table 1) presents another area for enhancement, especially when capturing high-speed motions like those in combat scenarios. This standard frame rate may not precisely capture the subtleties of such rapid movements, potentially leading to inaccuracies in our simulations. A plausible solution involves integrating additional cameras with slight temporal offsets, effectively elevating the frame rate [19] and thereby mitigating a possible source of error when trying to simulate fast-paced activities.

Alternatively, to enhance motion input quality, we could consider integrating our framework with alternative body tracking systems like OpenPose [30], which may offer more reliable captures for complex movements, as was suggested by Clark et al. [18].

## 5. Conclusions

This study introduced a novel, low-cost framework for simulating human motion in zero gravity, utilizing Microsoft’s Azure Kinect motion capture technology to generate authentic motion input. To demonstrate the tool’s effectiveness, we explored the biomechanical consequences of a weightless environment on human movement, specifically considering its implications for manned spaceflight. Our experiments encompassed a diverse range of motions, including rotations, locomotion, coordination, and martial arts movements, comprehensively exploring the proposed methodology.

Our findings suggest that many motions, including complex full-body coordination tasks, can be reproduced in zero gravity. However, these motions are bound by restrictions imposed by the conservation laws governing angular and linear momentum inherent in a weightless setting. The centroid of the body always remains fixed in location, adhering to the law of linear momentum conservation. At the same time, the ability to rotate is very limited due to the absence of external torques. Nevertheless, it is possible to achieve reorientation within the room through rotational movements by strategically employing motions that leverage the law of angular momentum conservation—a phenomenon also observed in diving. In conclusion, motions characterized by static postures and relying solely on the independent movement of the body’s extremities proved to be most effectively executed in our microgravity environment.

Given these constraints, most relevant fighting movements could be successfully simulated up to a certain precision, suggesting the feasibility of unarmed combat in a weightless environment. However, without any external surfaces, all movement is limited to a static state due to momentum conservation. In a space combat scenario, it would therefore be unattainable to perform the crucial action of moving towards or away from an opponent without applying external contact forces, like grabbing onto an object or another person.

Our study not only offers a preliminary understanding of how hand-to-hand combat could manifest in a microgravity setting, enriching our comprehension of potential challenges in future space scenarios, but more importantly holds potential implications for the development of tool-less exercise routines for astronauts. It therefore presents an effective framework for testing new approaches to maintain physical well-being in space.

While our proposed methodology has proven efficient and straightforward in simulating various motions in a weightless environment, certain limitations need acknowledgment. A remaining challenge lies in distinguishing the observable simulation effects actually caused by zero gravity, from potential errors introduced by the motion input, forward dynamics calculations, or the used model. Despite our best efforts, such as demonstrating the adherence of our results to prevailing conservation laws, validating the absolute realism of these simulations remains a formidable challenge. The inherent complexities of confirming the accuracy of the output motions underscore the need for further refinement of the simulation framework itself. Especially, the oversimplified PD controller of the forward dynamics calculations needs revision to address much of the shakiness observed in our current results.

In conclusion, our investigation of various motions and their possible manifestations in a space setting not only showcases an interesting application of motion capturing technology, but also lays the foundation for future research in related fields. By highlighting the practicality of our presented low-cost simulation framework, we aim to contribute to the preparation of upcoming space missions.

## Figures and Tables

**Table 1:** Operation details of the Azure Kinect DK depth sensor utilized in this research. The interested reader is encouraged to consult the hardware specifications [13] for further clarification.

Frame Rate	30 FPS
depth mode	narrow field-of-view (unbinned)
resolution	640×574
field-of-view	75°(h)×65°(v)
operating range	0.5 m to 3.86 m
exposure time	12.8 ms

**Table 2:** Summary of all recorded motions used for analysis. The columns include an index number, a brief description, the camera setting (wide/narrow, see Figure 6) yielding more stable motion input, and abbreviations indicating the movement’s relevance for the final evaluation (R: Rotation, L: Locomotion, C: Coordination, MA: Martial Arts). Terms related to martial arts in this table are exclusively derived from Karate.

Index	Name	Description	Setting	Relevancy
1	Arms	raising and lowering of both arms in a defencive manner, fixed stance	wide	MA
2	ArmsDefence	performing three standard defences, fixed stance	narrow	MA
3	ArmsRotFront	rotating both arms front ways (around pitch axis), fixed stance	narrow	R
4	ArmsRotSide	rotating both arms side ways (around pitch axis), fixed stance	wide	R
5	GodanArms	rotation of full body imposed by arm movement (from Kata Heian Godan)	wide	R, C
6	GodanJump	jump including 180-deg rotation of full body (from Kata Heian Godan)	wide	R, L, C
7	Punches	punches with alternating hands, fixed stance	narrow	MA
8	FightGyaku	fast back-hand punches from fighting position	narrow	MA
9	FightKizame	fast front-hand punches from fighting position	wide	MA
10	Fight	alternating front and back-hand punches from fighting position	narrow	MA
11	HeianSandan	full body coordination task (from Kata Heian Sandan)	wide	C
12	Jion	full body coordination task (from Kata Jion)	narrow	C
13	Hips	turning of hips from side to front and back in fixed stance	narrow	R, C
14	RotatingBody	sweeping arms causing repeated body rotations of 180 degrees, lunge stance	narrow	R, C
15	Jump	repeated two-legged jumps with knees drawn up high	narrow	C
16	StepKiba	side step in horse riding stance (Kiba Dachi)	narrow	L
17	StepKokutsu	front step in back stance (Kokutsu Dachi)	wide	L
18	StepZenKutsu	front step in front stance (Zen Kutsu Dachi), with attack and defence motion	narrow	L, MA

## Data Availability

All computer code accompanying our research and all data stemming from the motion experiments, integral to the evaluations, are available as Supplementary Materials to this publication.

## Supplementary Materials

The C++ source code essential for deploying our simulation framework, and all motion data (including videos) used in our study can be downloaded at https://www.mdpi.com/article/10.3390/s24061770/s1. The provided source code includes the directories: model (for generating the OpenSim model), record (for creating synchronized recordings with multiple Azure Kinect DK devices), and simulation (for employing the simulation framework).

## Abbreviations

The following abbreviations are used in this manuscript:

3D	three-dimensional
DK	developer kit
FPS	frames per second
MP	megapixel
PD	proportional-derivative
RGB	red-green-blue
